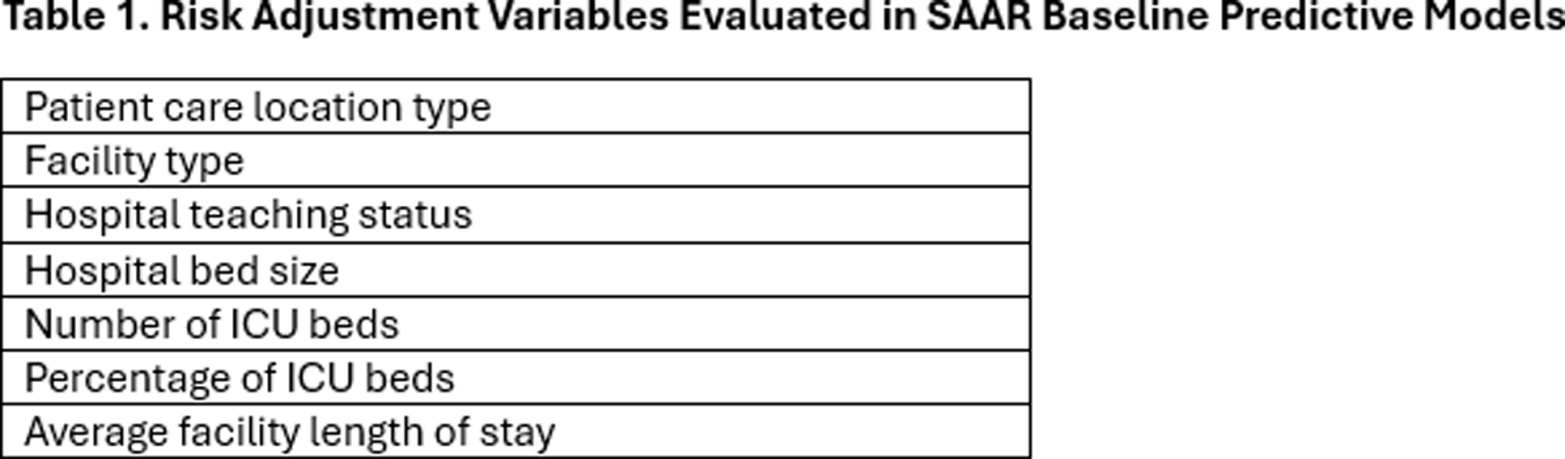# 83 Evaluating Barrier Integrity of Tyvek-Packaged Sterile Supplies Following Humidity and Moisture Exposure

**DOI:** 10.1017/ash.2026.10509

**Published:** 2026-06-23

**Authors:** Kranthi Swaroop Koonisetty, Qaiser Jahan, Teah Snyder, Ryan Bariola

**Affiliations:** 1 Pennsylvania Department of Health; 2 Division of Healthcare Infection and Prevention, Pennsylvania Department of Health

## Abstract

**Background:** A limitation of National Healthcare Safety Network’s (NHSN) Standardized Antimicrobial Administration Ratio (SAAR) is incorporation of only facility and location-level characteristics in predictive antimicrobial use (AU) models (Table 1). Facilities with more medically complex patients may question SAAR’s applicability to their facilities due to lack of patient-level characteristics in predictive models. Case-mix index (CMI), a weighted average of a facility’s patient acuity, could be a marker of patient-level severity for comparison purposes. We investigated differences in SAAR values between facilities with different CMIs. **Methods:** We stratified Pennsylvania acute care hospitals (ACH) by CMI to compare median SAAR values between hospitals within each CMI quartile. We used 2024 SAAR values from adult inpatient locations using NHSN’s 2017 baseline predictive models. For CMI, we used Centers for Medicare and Medicaid Service’s (CMS) 2024 Diagnosis-Related Group at the hospital CMS Certification Number (CCN[KK1] ) level. CMI is defined at the CCN level while AU is reported under multiple NHSN organization IDs (orgIDs); therefore, observed and predicted antimicrobial days were summed across all orgIDs within each CCN to generate a single CCN-level SAAR for Adult All-Antibacterials. Hospitals were grouped into quartiles based on CMI and median SAAR values were calculated for each group. SAAR differences across CMI quartiles were assessed using the Kruskal–Wallis test. **Result:** Among 112 Pennsylvania CCN-level ACHs with complete AU and CMI data, hospitals were evenly distributed across CMI quartiles (N = 28 each; CMI range 1.26–2.88). Median SAAR values were similar across quartiles (Q1=0.96, Q2=0.97, Q3=0.92, Q4=0.92). SAAR did not differ significantly across quartiles (Kruskal–Wallis χ² = 1.94, p = 0.58). Boxplots showed substantial overlap in SAAR distributions across quartiles (Figure 1). Conclusion Median SAAR values did not differ significantly across CMI groups. Facilities serving more medically complex patients should not discount the SAAR based on lack of inclusion of patient-level characteristics in the predictive models. More research is needed to clarify if currently included SAAR predictive model variables are adequate proxies for patient acuity and if inclusion of additional patient-level factors would produce more accurate predictions. Our CMI dataset only included publicly available Medicare data, which may limit generalizability across age groups

**Figure:**
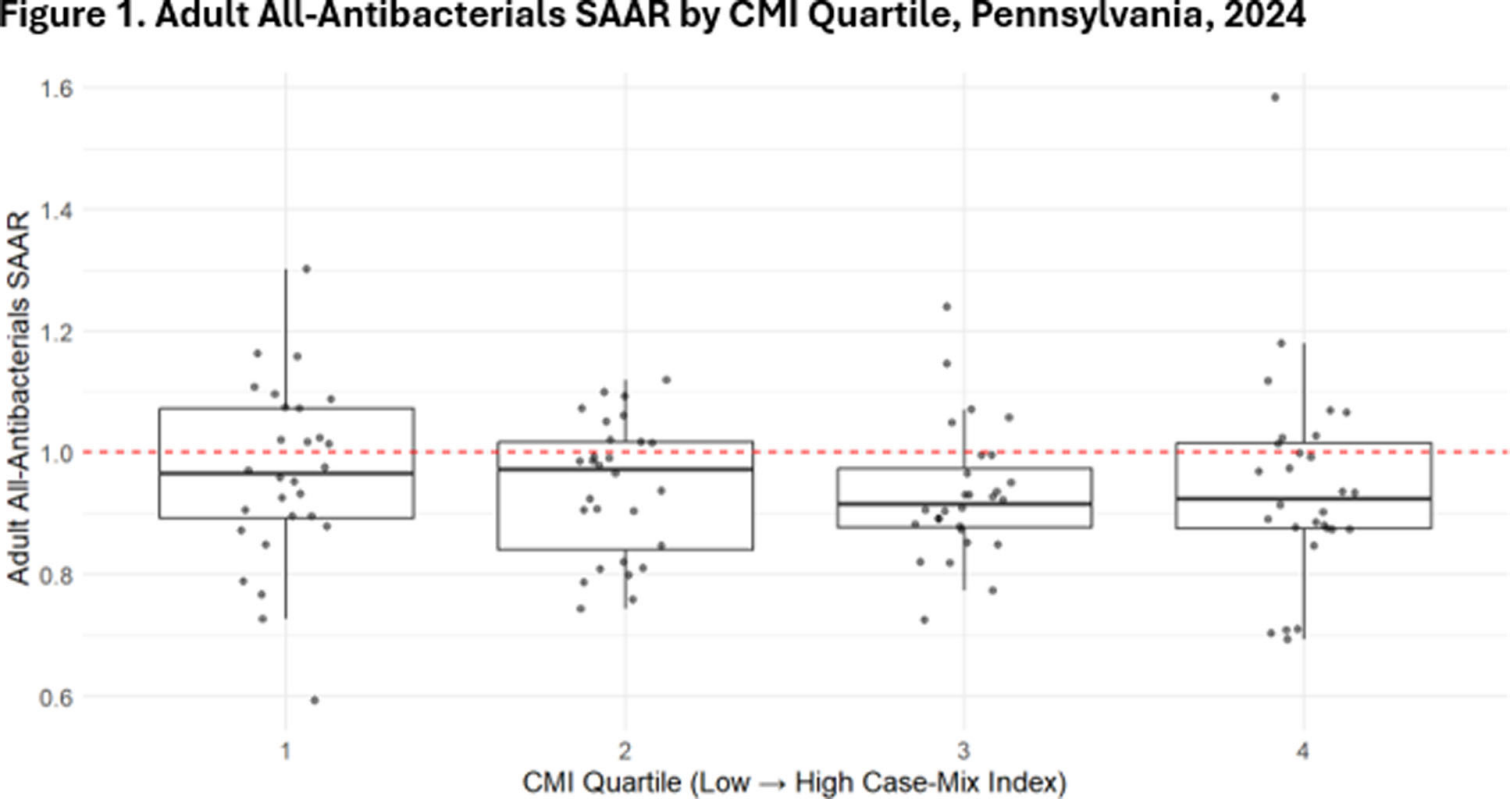

**Figure:**